# Energetic cost of weight shifting and asymmetric weight-bearing during standing in humans

**DOI:** 10.1152/japplphysiol.00294.2026

**Published:** 2026-08-27

**Authors:** Matto Leeuwis, Ralf Rienks, Maarten A. Frens, Johan J.M. Pel, Patrick A. Forbes

**Affiliations:** 1 Department of Neuroscience, Erasmus MC, University Medical Center Rotterdam, Rotterdam, The Netherlands; 2 Department of Biomechanical Engineering, Delft University of Technology, Delft, The Netherlands

**Keywords:** Standing balance, Postural asymmetry, Energy expenditure, Metabolic cost, Behavioral energetics

## Abstract

During prolonged standing, humans commonly shift their weight and spontaneously alternate between symmetric and highly asymmetric standing, bearing most of their body weight on one leg. Here, we tested the hypothesis that humans adopt asymmetric weight-bearing to reduce metabolic cost by exploiting the sublinear scaling of muscle activation maintenance costs predicted by metabolic models. We measured energy expenditure using indirect calorimetry while participants (*N* = 20) completed three randomized standing conditions: (i) static symmetric standing, (ii) symmetric standing with periodic lateral weight shifts, and (iii) asymmetric standing with periodic switching of the supporting leg. Weight shifting every 30 s from an otherwise symmetric stance increased overall energy expenditure by 5.3% [95% CI: 2.4%, 8.3%] relative to static symmetric standing. Maintaining an asymmetric posture while shifting to the other leg every 30 s increased energy expenditure by a further 7.0% [3.5%, 10.4%], contrary to our hypothesis that asymmetry would reduce metabolic cost. We additionally measured postural variability and plantarflexor muscle activity to explore potential mechanisms underlying energetic differences. Asymmetric weight-bearing increased postural variability relative to symmetric standing, with the average mediolateral upper-body velocity increasing by 118% [90%, 147%], suggesting that an increased stabilization effort contributed to the cost. Muscle activity in the weight-bearing leg strongly increased for the medial gastrocnemius, while soleus activity increased less than predicted. These findings demonstrate that weight shifting and asymmetric weight-bearing, two common standing behaviors, may not reflect behavioral energetic optimization and instead originate from non-energetic objectives such as alleviating discomfort or fatigue.

## Introduction

When standing for prolonged periods, such as waiting for the bus or standing in line at a store, humans shift between symmetric and highly asymmetric postures, resting most of their body weight on a single leg. Postural asymmetry is so characteristic of natural human standing that it has been repeatedly depicted in the arts across cultures and historical periods, known in classical arts as contrapposto (e.g., Michelangelo’s David or the Venus of Milo), in which the supporting leg bears the body weight while the pelvis and trunk counterrotate. This everyday behavior was quantified during prolonged 16-minute experimental trials (1), where a bimodal preference emerged for symmetric (i.e., ~50% of body weight on each leg) and asymmetric weight-bearing postures with asymmetry averaging around 74% (i.e., 87% of weight on one leg and 13% on the other). This preference was stronger among female participants, who spent 37% of the duration in highly asymmetric postures, compared with 14% among male participants (1). The reason humans use uneven weight distribution is unclear, though a commonly held hypothesis is that weight-shifting alleviates discomfort and fatigue in the postural muscles, joints, and feet (2–4). This hypothesis is supported by the observation that prolonged standing (>10 minutes) increases the incidence of weight shifts (1, 5) and postural variability (6, 7).

Here, we consider these behaviors from a metabolic efficiency perspective. Humans broadly optimize energy expenditure in walking (8–17), prefer minimal-cost postures during symmetric standing (18), and minimize effort when recovering from postural perturbations (19). Asymmetric weight-bearing might allow participants to further reduce energy expenditure by efficiently utilizing plantarflexor muscle physiology. Muscle metabolic models predict that the energetic cost of maintaining constant muscle activation during isometric contraction, the main contributor to the energetic cost of standing (18), scales to a sublinear power of 0.6 with activation (20–22). This sublinear power was derived from the relationship between muscle heat generation and force in the human vastus lateralis (23) and gastrocnemius medialis (24). In these studies, low force production, approximately 10% of maximum voluntary contraction (MVC), exhibited up to sixfold less efficient force-heat relationship than higher force levels of 50-90% MVC (23). This reduced efficiency at low force levels likely reflects the energetic cost of repeated force generation and relaxation cycles in motor units firing at low, unfused frequencies (23, 24). With a power of 0.6, doubling muscle activation multiplies muscle energy expenditure by only ×1.52. During bipedal standing with symmetric foot placement, shifting most of the weight to one leg may allow that leg to generate most of the plantarflexion moment required to counteract gravity while relaxing the other. Redistributing weight to one leg could potentially save up to 24% of activation maintenance costs in the plantarflexor muscles (one leg at two units of activation: 2^0.6^ = 1.52, as opposed to two legs at one unit of activation: 2 ⋅ 1^0.6^ = 2).

On the other hand, Asymmetric weight-bearing also alters hip abduction moments (25, 26) and increases postural variability (1, 27), potentially increasing the energetic demand of muscles and control processes beyond the ankle. Previous work has shown that the energetic cost of standing increases by ~24% or more when postural variability is experimentally increased through a tandem stance or balance board (28), or by up to ~9% when participants are asked to maintain their center of pressure within a narrow range while standing on a compliant surface (29). Similarly, in an exploratory experiment with 7 participants, Miles-Chan et al. (30) reported an average energetic cost for experimentally prompted lateral weight shifts that was ~6% higher than relaxed standing, although this comparison was not statistically tested. At present, it is unknown how energy expenditure changes with asymmetric weight-bearing and whether the energetic savings predicted from plantarflexor muscle physiology outweigh other potential costs associated with postural variability and joint loading.

In this study, we evaluated the energetic cost of two common behaviors during standing: periodic weight shifting and asymmetric weight-bearing. Twenty participants performed three conditions in random order: Baseline, static symmetric standing; Shifting, symmetric standing with brief lateral weight shifts every 30 s; and Asymmetric, asymmetric weight-bearing with weight shifts from one leg to the other every 30 s, while remaining in an asymmetric posture between shifts. We hypothesized that asymmetric weight-bearing between weight shifts reduces energy expenditure compared with standing symmetrically between weight shifts (Asymmetric vs. Shifting), while weight shifts incur a small energetic cost relative to static symmetric standing (Shifting vs. Baseline). Motivated by sex differences in the incidence of asymmetric postures (1), we compared the magnitude of postural asymmetry in the Asymmetric condition and included sex as a between-participant factor when comparing energy expenditure to rule out potential confounds. We additionally examined postural variability, lean and knee angles, and plantarflexor muscle activity as secondary outcomes to investigate potential mechanisms underlying energetic differences.

## Methods

### Participants

A total of 20 healthy adults (sex assigned at birth: 9 male, 11 female; age = 28.0 ± 5.8 years (mean ± standard deviation, range 19-48 years old); height = 179.4 ± 11.4 cm; mass = 74.5 ± 15.1 kg) participated in the study. The study was approved by the Erasmus Medical Center ethics committee (MEC-2023-0205) and conducted in accordance with the Declaration of Helsinki. Participants received the information letter for the experiment at least 48 hours before participating and provided written informed consent for their participation and the optional permission to publish the coded data collected during the experiment under a Creative Commons Attribution (CC BY) license (31). The following exclusion criteria were enforced: history of back or neck pain, pain during standing or walking, balance problems or use of balance aids, pregnancy or current breastfeeding, neuromuscular injury, or use of acute or chronic psychoactive medication (including ADHD medication). The target sample size of 20 was selected to match comparable within-subjects studies assessing the energetic cost of postural variability and weight shifting that performed paired comparisons across 10-19 participants (28, 29). An additional male participant, not listed in the group characteristics, performed the experiment but was excluded because their postural movement during the Shifting condition deviated from the instructed symmetric stance, drifting between symmetric and asymmetric postures near the edges of the target and exhibiting a CoP distance from the mean (6.50 mm) equal to mean plus ~9 times the standard deviation (1.73 ± 0.54 mm) of the remaining participants and far exceeding the next highest participant (3.22 mm).

### Protocol

Participants were tested in either a morning (10:00, *N* = 6) or afternoon (14:00, *N* = 14) session. As caloric and caffeine intake may influence metabolic assessments (32), participants were asked to fast for 3 hours preceding the experiment for food and caffeine. During preparation, basic anthropometric measurements were collected from all participants, including height, ankle height at the lateral malleolus, and hip width at the greater trochanters. Body mass was collected using the force plate (see Experimental measurement setup). The foot placement on the force plate was standardized by positioning the centers of the ankle joints at hip width, estimated as 0.532 times the measured distance between the greater trochanters (33). The center of rotation of the ankle joint was assumed to lie at the anterior edge of the medial malleolus (34) and was aligned with the midline of the force plate.

Most participants in this experiment (7 male, 9 female) first completed 7 trials lasting 3 minutes for another experiment in the same session, which involved quiet standing with electrical vestibular stimulation or an immobilizing backboard (35) to modulate postural variability and measure energy expenditure (trial average <1.70 W/kg across all conditions). All participants were seated for at least 3-5 minutes before the start of data collection in the current study (either during preparation or as a planned break following the other experiment). The chair was placed directly beside the force plate to limit movement. Participants were not allowed to walk around during preparation or breaks, ensuring they did not perform any physical activity beyond sitting or standing for approximately 30 minutes before trials started.

**Figure 1:**
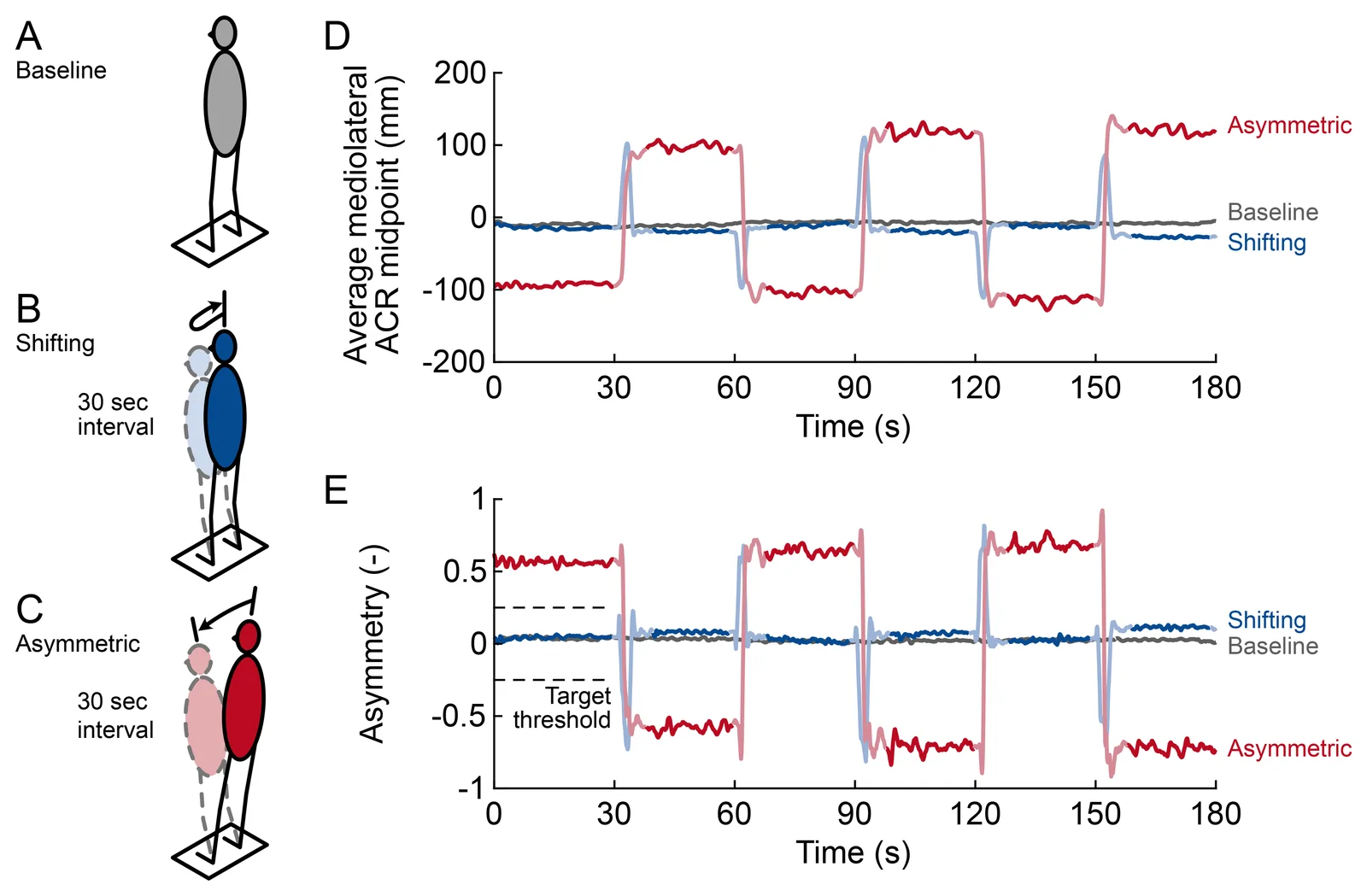
Schematic overview of the experiment and representative behavior during standing in each condition. A: Baseline: participants stood in a static symmetric stance. B: Shifting: participants stood symmetrically but were prompted every 30 s to briefly shift their weight laterally to alternating sides and return to a symmetric stance. C: Asymmetric: participants adopted a preferred asymmetric stance and switched their supporting leg every 30 s following a prompt. Periods of weight shifting in the Shifting and Asymmetric conditions were excluded from analysis (muted color at 30 s increments). D: Representative participant data of the midpoint position between both shoulder markers (acromion - ACR) in the mediolateral direction, showing the position of the upper body during static standing and prompted weight shifts. E: Representative participant data of the asymmetry metric (Eq. 1), indicating how asymmetric the center of pressure was positioned compared to the stance width. An asymmetry of 0 represents a symmetric weight distribution, whereas ±1 indicates full loading on the left (-1) or right (+1) leg. The threshold of the auditory target is indicated with black dashed lines. Colors match conditions: Baseline (gray), Shifting (blue), Asymmetric (red).

Participants performed three conditions in a randomized order while standing on the force plate, each lasting 3 minutes. This duration was selected to leave a 2-minute analysis window for energy expenditure after excluding the first minute to remove possible transient effects (18), leaving an analysis window comparable to previous work (28, 29). Trials were separated by 110 ± 64 s of standing while the next condition was explained. In the Baseline condition (Figure 1A), participants stood quietly with their weight evenly distributed between the feet. In the Shifting condition (Figure 1B), participants stood symmetrically and received periodic prompts at 30 s intervals to briefly shift their weight to one side before returning to a symmetric posture. This condition quantified the energetic cost of periodic weight shifts relative to static symmetric standing in Baseline. In the Asymmetric condition (Figure 1C), participants were asked to stand while supporting more weight on one leg than the other and were prompted at 30 s intervals to shift their weight to their other leg, matching the Shifting condition. The recording began with participants already in an asymmetric stance. The Asymmetric condition combines the energetic contributions of weight shifts and asymmetric weight-bearing; comparison with the Shifting condition allowed us to identify the energetic cost associated with asymmetric standing while controlling for the cost of the weight shift itself. The 30-second interval for weight shifting was selected to approximate the average frequency with which people spontaneously shift their weight during prolonged standing (1) and to avoid potential discomfort associated with continuously maintaining most of the body weight on one leg, which we observed during pilot testing. To ensure that participants maintained a sufficiently asymmetric posture, a sound cue was played if the lateral distance between the midline and center of pressure was less than 1/8^th^ of their total stance width (i.e., an asymmetry of ~0.25; see Eq. 1 and Figure 1E). This threshold was selected to split the bimodal weight distribution preference reported by Prado-Rico and Duarte (1) into its symmetric and asymmetric components, allowing participants to freely select any asymmetry beyond the threshold. Similarly, the target was used during the Baseline and Shifting conditions to maintain the center of pressure within 1/8^th^ of the total stance width. Representative motion capture and weight-bearing asymmetry data of a single participant are shown in Figure 1D-E.

### Experimental measurement setup

We collected motion capture data using reflective markers placed on anatomical landmarks according to the simplified marker set by Tisserand et al. (36), with the addition of 4 markers on the second metatarsal and calcaneus (bilaterally) and exclusion of the sacral marker (16 markers total). The motion capture system consisted of 8 cameras running at 100 Hz (4 Miqus M3 and 4 Miqus M5, Qualisys Track Manager v2024.2, Qualisys AB, Göteborg, Sweden), together with an analog measurement board (Qualisys Analog Interface 16 Channels, 230597, Qualisys AB, Göteborg, Sweden) for electromyography (EMG) measurements at 1000 Hz, a sync box (Qualisys Sync Box, 410850, Qualisys AB, Göteborg, Sweden), and a single force plate recording force and moments at 1000 Hz (Plate: BMS 400600HF-1K, Amplifier: Optima OPT-SC, AMTI, MA, USA). A single force plate was used due to equipment constraints; therefore, individual limb loading was estimated using a previously validated ground reaction force splitting algorithm (18). A TV was placed 3.2 m in front of the participant to present a textual cue paired with an auditory tone at 30-second intervals, prompting them to shift their posture to the side and back (Shifting condition) or to switch leaning side (Asymmetric condition). This prompt was timed and delivered using a custom MATLAB script utilizing QTM Connect for MATLAB v2024.2.

Muscle activity was measured bilaterally from the soleus (Sol) and gastrocnemius medialis (mGas). EMG signals were amplified using a pre-amplifier, high-pass filtered through an analog filter block (NL844 Preamplifier (gain x1000, high-pass filter 30 Hz), NL820A Isolator (gain x2), and NL135 Filter (low-pass filter 500 Hz), Neurolog, Digitimer, UK), and recorded at 1000 Hz with a 16-bit resolution over a ±10V range on the analog measurement board connected to the motion capture system. The ground electrode was connected to the right ankle on the lateral malleolus. Electrodes were placed following the SENIAM 8 guidelines (37).

Metabolic energy expenditure was measured throughout the experiment using a metabolic analyzer (system: MS-CPX, Carefusion Germany, Hoechberg, Germany; wearable analyzer: SBx/CPX, CareFusion Germany, Hoechberg, Germany; mask: V-982185, Vyaire Medical, USA), which stored breath-by-breath data. The analyzer computed energy expenditure using the Brockway equation (38). The manufacturer-reported accuracy of the system is 2% or 0.05 L/min for ventilation and 3% or 0.05 L/min for O_2_ and CO_2_ uptake (MS-CPX Instructions for Use Version 01.00). Before each session, the device was calibrated using the MS-CPX automated calibration programs, which included a flow calibration and a gas analyzer calibration with a prescribed gas mixture (15.94% O_2_, 5% CO_2_, V-892587, Vyaire Medical, USA). Metabolic analyzer measurements were synchronized to motion capture data by manually starting the measurements at the same time.

### Data analysis and statistics

Metabolic energy expenditure, ground reaction forces, EMG, and motion capture signals were analyzed offline using custom MATLAB scripts (R2023b, MathWorks, MA, USA). The first minute of calorimetry data was omitted in each trial to ensure that participants had reached steady-state energy expenditure (18). Metabolic energy expenditure was then computed by summing breath-by-breath expended energy during complete breaths within this window and dividing by the sum of breath durations, yielding a time-averaged energy expenditure for each trial. The average rate of energy expenditure was subsequently divided by body weight and reported as weight-normalized metabolic power (W/kg).

Weight shifts following a prompt in the Shifting and Asymmetric conditions were excluded from all metrics except energy expenditure, owing to the long time constant of indirect calorimetry (39). These periods were identified as samples in which the average lateral marker velocity of the ACR markers exceeded 60 mm/s, and included a lead time of 2 seconds before the first sample exceeding this threshold and a lag time of 5 seconds after the last sample exceeding it. The velocity threshold and lead/lag times were selected after data collection through visual inspection to ensure that transition movements were captured within this window. For each weight shift, the average duration the velocity threshold was exceeded was 2.76 ± 0.63 s for Shifting and 1.53 ± 0.69 s for the Asymmetric condition (excluding the lead and lag time).

Weight distribution asymmetry was computed based on the ground reaction forces under each foot:

(1) $$\text{asymmetry}\;=\frac{F_{z,\;\text{Right}\;}-F_{z,\;\text{Left}\;}}{F_{z}},$$

where the difference between ground reaction forces on the left and right foot is divided by the total ground reaction force (1). An asymmetry of -1 or +1 corresponds to supporting all weight on the left or right leg, respectively. The forces were estimated using a ground reaction force splitting algorithm, which assumes that each foot’s center of pressure must lie on the line between the calcaneus and second metatarsal (18). The CoP of each foot was estimated by projecting the anteroposterior coordinate of the net CoP onto the midline of the feet. Using the assumed foot CoP locations, the vertical force *F*_*z*_ under each foot was proportionally distributed such that the moment around the net CoP was zero. This algorithm was previously reported to have a 95^th^ percentile absolute error of *e =* 18.6 N per foot across all samples from 151 trials of weight shifts in the dual-stance phase of gait initiation (18). Using Eq. 1, adding or subtracting this error to the left or right side would correspond to an asymmetry error (2*e* / *F*_*z*_) of ~0.05 for a person with the mean mass of the included participants (74.5 kg).

The lean angle of the center of mass with respect to gravity was analyzed to ensure it did not affect energy expenditure across conditions (18). Lean angle was estimated from the CoP by assuming the center of mass was directly above the CoP on average throughout the trial and that the small-angle approximation could be applied. CoP data were low-pass filtered (4^th^ order zero-phase Butterworth, cutoff frequency 20 Hz) to minimize the effect of measurement noise. The center of mass height with respect to the ankle joint was estimated by multiplying the body height by 0.56 for female and 0.57 for male participants (40), and subtracting the ankle height. Lean angle was then estimated in radians by dividing the anterior-posterior distance of the CoP with respect to the ankle joint by the center of mass height:

(2) $$\text{lean}\;\text{angle}\;=\frac{CoP_{\text{AP}}}{h_{\text{CoM}}-h_{\text{ankle}\;}}.$$

Postural variability was quantified using two metrics: (i) the average absolute distance of the CoP from the average location of the CoP in the segment, which previous work suggests correlates with energetic cost (28, 29), and (ii) the average velocity magnitude of both acromion (shoulder) markers to quantify bodily motion. The CoP distance for each segment between transitions was computed as the mean distance of the CoP from the segment mean. Data from each segment within each condition were then averaged to compute the participant means. The Baseline condition was divided into 30-second segments to avoid bias introduced by differences in segment length. Marker velocity was computed as the mean velocity in the anteroposterior (AP) or mediolateral (ML) direction of both acromion markers throughout the valid segments between transitions. Marker trajectory data were preprocessed in Qualisys Track Manager (v2024.2), allowing a prediction error of up to 20 mm and a residual of 5 mm during 3D tracking. Gaps ≤100 ms in marker data were filled via polynomial interpolation; remaining gaps (0.02% of samples total) were omitted from analysis. Marker trajectories were low-pass filtered in MATLAB (4^th^ order zero-phase Butterworth, cutoff frequency 6 Hz) before time derivatives were computed to obtain marker velocity.

We estimated the knee angle as the sagittal-plane-projected angle between the ankle (lateral malleolus), knee (lateral femoral condyle), and hip (anterior superior iliac spine) motion capture markers. We then examined changes in the knee angle across conditions to address the possibility that lower-limb kinematic differences could affect muscle activation or energy expenditure. Knee angle was reported separately for the loaded and unloaded legs in the Asymmetric condition to check whether participants overextended (e.g., “locked”) their knee joint more compared to Baseline.

Raw EMG recordings were high-pass filtered (4^th^ order zero-phase Butterworth, cutoff frequency 10 Hz), full-wave rectified, and then low-pass filtered (4^th^ order zero-phase Butterworth, cutoff frequency 100 Hz). These signals were then normalized per muscle for each participant to their maximal activation across all trials. In the Asymmetric condition, EMG segments were partitioned according to which leg was currently the loaded or unloaded leg, and activation averages were computed using equal left and right-leaning durations to minimize the effects of normalization differences between legs. For the other conditions, the left and right EMG were combined in participant averages. Based on the asymmetry magnitude reported during natural standing (~0.75, (1)), a proportional redistribution of plantarflexion moment would result in approximately 75% greater activation in the loaded leg and 75% reduced activation in the unloaded leg relative to Baseline. In one participant, the end of the EMG recording was missing due to a hardware error (S06, Shifting, 59.5 s missing); averages were computed over the valid periods in the remaining 120.5 s of data.

Statistics were performed in JASP 0.18.1 (41) with *α* = 0.05. A repeated-measures ANOVA was performed with Condition (Baseline, Shifting, Asymmetric) as a within-subjects factor to test for differences in metabolic energy expenditure, asymmetry, lean angle, CoP distance, and ACR marker velocity. Sphericity was assessed using Mauchly’s test, and a Greenhouse-Geisser correction was applied if sphericity was violated. If a significant main effect was found, post hoc comparisons were conducted in JASP using a Holm correction. The two primary hypothesis-driven comparisons were Baseline vs. Shifting and Shifting vs. Asymmetric; the remaining comparison (Baseline vs. Asymmetric) is reported for completeness. For energy expenditure, sex was additionally included as a between-subjects factor to assess whether it confounded the primary energetic results. To assess whether energy expenditure varied systematically with chronological trial order, we fit a linear mixed-effects model in MATLAB with trial order as a fixed effect and participant as a random intercept. We performed an independent-samples *t*-test to compare the magnitude of preferred postural asymmetry between male and female participants in the Asymmetric condition, motivated by prior evidence of sex differences in the incidence of asymmetric postures (1). For the secondary EMG and knee angle analyses, the Asymmetric condition was split into the loaded and unloaded legs, yielding 4 conditions for the repeated-measures ANOVA (i.e., Baseline, Shifting, Asymmetric-loaded, and Asymmetric-unloaded). Post-hoc reporting for these ANOVAs was limited to three contrasts: Baseline vs. Shifting, Baseline vs. Asymmetric-loaded, and Baseline vs. Asymmetric-unloaded; the remaining pairwise comparisons are available in the supplementary data and code for completeness. Data are presented as mean ± SD, and mean differences between conditions with unadjusted 95% CI [lower, upper]. Degrees of freedom were reported as *t*(df) and *F*_(df1, df2)_, and listed in decimals if a Greenhouse-Geisser correction was applied.

## Results

### Weight shifting and asymmetric weight-bearing increase energy expenditure

**Figure 2:**
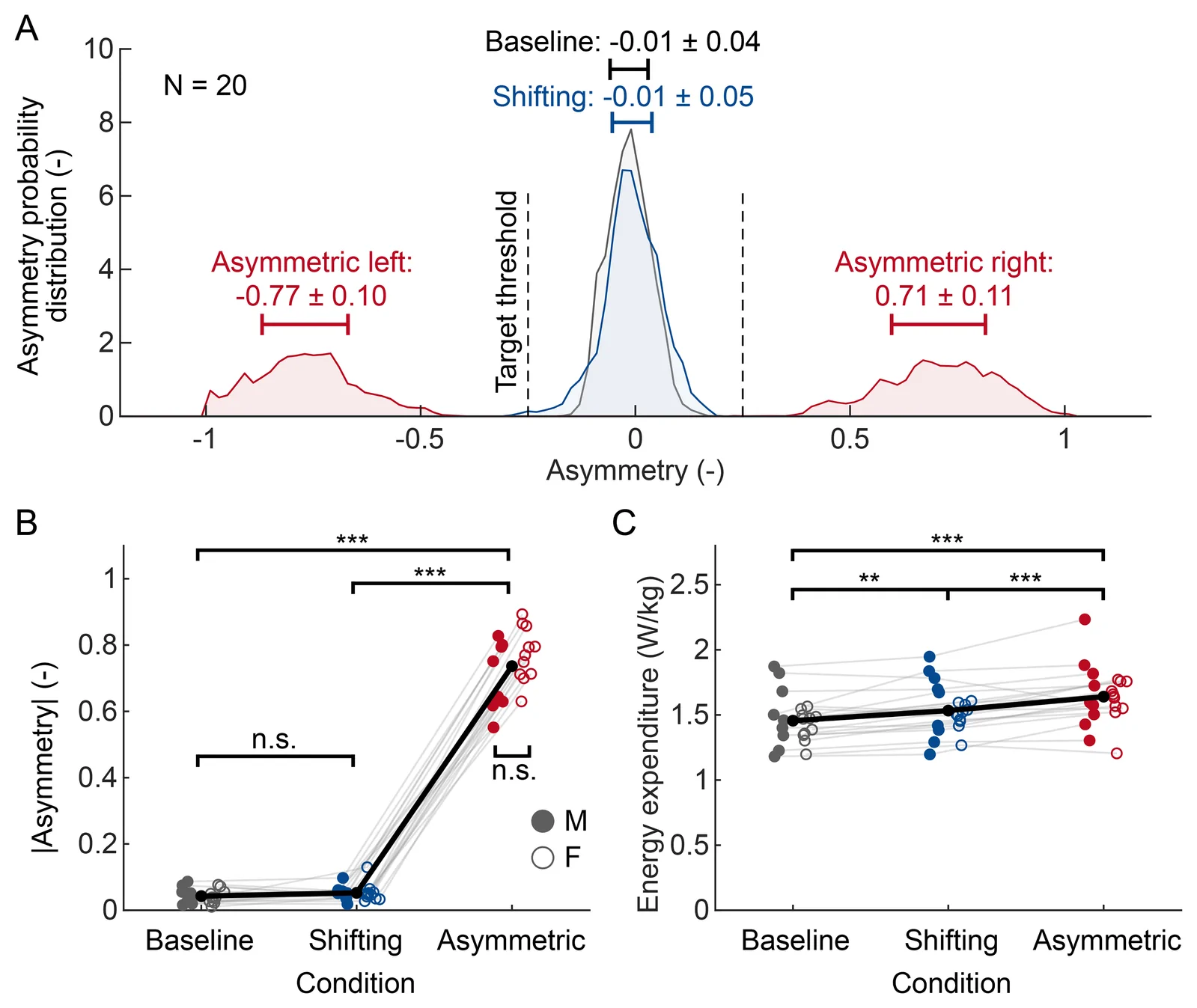
Overview of asymmetry and energy expenditure in the three standing conditions. A: Probability distribution of the asymmetry metric during the experimental conditions, constructed using pooled samples excluding prompted weight shifts from all participants. The distribution depicts the variation in postural asymmetry during the static standing periods across experimental conditions, excluding the transitions. Positive asymmetry reflects a right-leaning posture. Distributions are normalized such that their integral equals 1. Values above peaks are mean ± SD of the participants’ average asymmetry in that condition. Means and SD of the Asymmetric condition were split between the left and right-leaning postures. Vertical dashed lines indicate the asymmetry thresholds for the auditory target. B: Average absolute asymmetry of the weight distribution over the conditions. The additional statistical annotation on absolute asymmetry in the Asymmetric condition represents an independent-samples *t*-test comparing male and female participants. C, Average energy expenditure of all participants from indirect calorimetry measurements. Male and female participants are indicated with closed and open circles, respectively. The black line depicts the condition average. Gray connecting lines represent individual participant averages. Statistical annotations indicate the result of post-hoc comparisons. Labels: n.s.: not significant, *: *p* ≤ 0.05, **: *p* ≤ 0.01, ***: *p* ≤ 0.001. M: male, F: female.

We first assessed postural asymmetry across conditions in which participants stood symmetrically (Baseline, Figure 1), symmetrically with intermittent weight shifts (Shifting), or asymmetrically while alternating the support leg (Asymmetric). We found a significant main effect of condition on our asymmetry metric (Figure 2A-B, Table 1, Eq. 1). In the Baseline and Shifting conditions, participants were instructed to maintain a symmetric posture and had a low absolute asymmetry that did not differ between the conditions (Figure 2B, Table 1, difference: 0.01 [-0.004, 0.023]). In contrast, in the Asymmetric condition, participants were instructed to self-select a posture that placed more weight on one leg than the other, greatly increasing asymmetry compared to the Baseline and Shifting conditions (Figure 2B, Table 1). The comparison of preferred postural asymmetry between male and female participants showed no difference (Male: 0.69 ± 0.10, Female: 0.77 ± 0.08, difference: 0.08 [−0.01, 0.16], *t*(18) = 1.90, *p* = 0.073, Figure 2B).

We next evaluated how periodic weight shifts (Baseline vs. Shifting) and asymmetric weight-bearing (Shifting vs. Asymmetric) affect the energetic cost of standing. There was a main effect of Condition on energy expenditure (Figure 2C, Table 1) with differences between each of the three conditions. Energy expenditure increased between the Baseline and Shifting conditions by 0.08 W/kg [0.03, 0.12] (i.e., 5.3% [2.4%, 8.3%]), indicating that periodic weight shifts impose an energetic cost increase relative to standing in a static symmetric posture. We estimated this cost to be 2.3 J/kg [1.0, 3.6] per transition by dividing the additional energy expended per minute with respect to Baseline by the frequency of weight shifts (2 per minute). The Asymmetric condition further increased energy expenditure by 0.11 W/kg [0.05, 0.16] (i.e., 7.0% [3.5%, 10.4%]) compared to the Shifting condition (Figure 2C, Table 1), indicating that an asymmetric weight distribution between shifts required more energy than a symmetric stance. The primary energetic results did not depend on sex, as neither the main effect of sex nor the condition × sex interaction reached significance (Table 1, note ^a^). Lean angle (Eq. 2) did not vary across conditions (Table 1), indicating that it did not confound observed energetic differences (18). The standard error of the pairwise differences (0.03 W/kg) was small compared to the differences between conditions (≥0.08 W/kg), indicating that random sources of variability, such as behavior or measurement system noise, had limited influence on these differences. A linear mixed-effects model testing for a trend in energy expenditure across chronological trial order (independent of condition) showed no significant slope (slope = -0.03 W/kg [-0.07, 0.01] per trial, *t*(58) = -1.61, *p* = 0.113).

### Asymmetric weight-bearing increases postural variability

**Figure 3:**
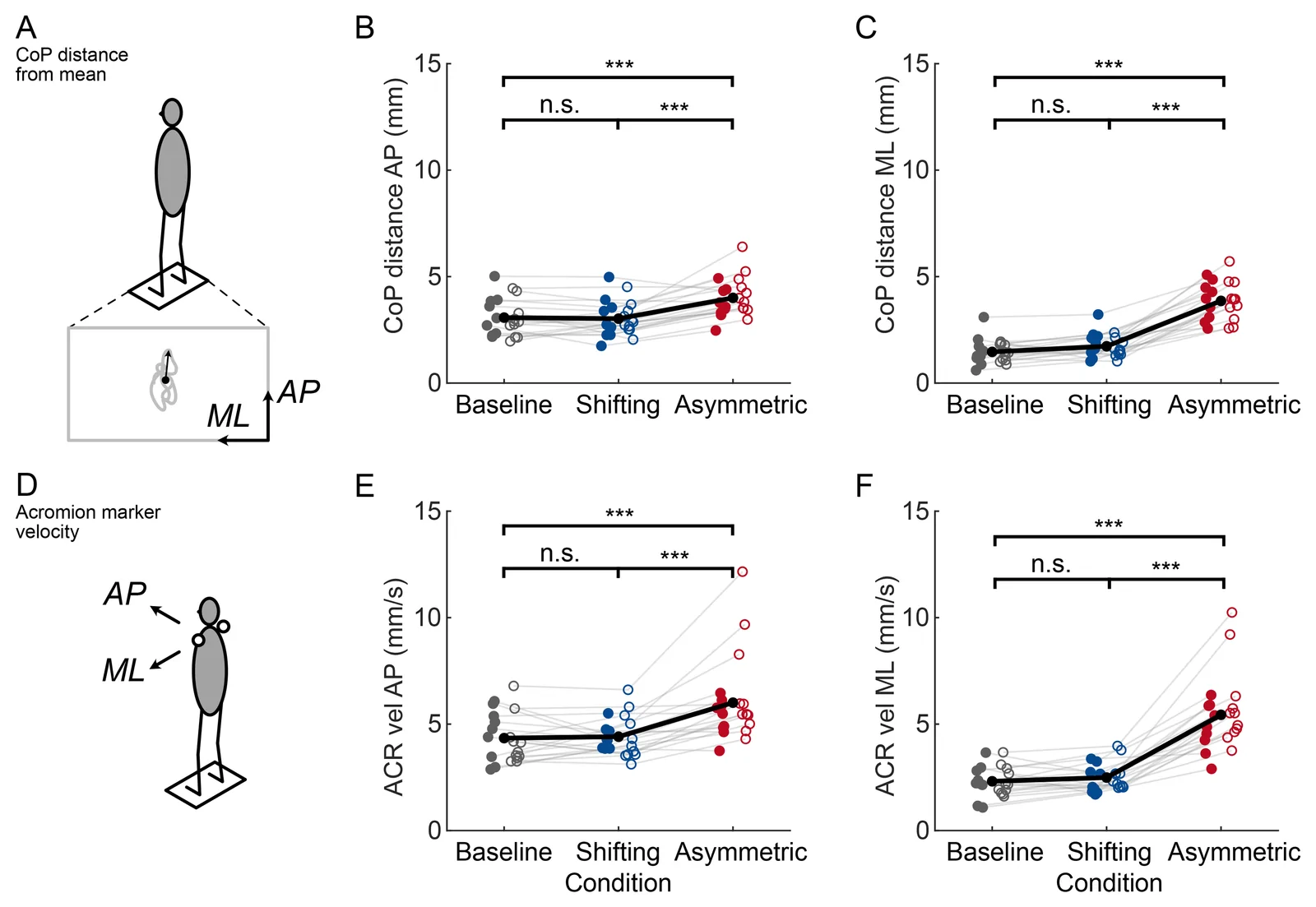
Asymmetric weight-bearing increases variability during static standing between shifts. A: Schematic representation of the average distance of the CoP with respect to the average position of that trial (28, 29). Distances were computed during the periods between weight shifts (Shifting and Asymmetric conditions) and then averaged per participant across periods. B-C: Mean CoP distance in AP and ML direction. D: Schematic representation of average absolute velocity of the acromion (shoulder) markers. Velocity was computed for the left and right markers separately and then averaged for each participant. E-F: Mean ACR velocity in AP and ML direction. Statistics and group means are provided in Table 1. Gray connecting lines represent individual participant averages. Labels: n.s.: not significant, *: *p* ≤ 0.05, **: *p* ≤ 0.01, ***: *p* ≤ 0.001, M: male, F: female, AP: anteroposterior, ML: mediolateral.

We next examined whether the higher energetic cost in the Asymmetric condition may result from differences in postural variability, quantified using (i) the average distance of the CoP from its mean position (Figure 3A-C), and (ii) the average velocity of both acromion markers (Figure 3D-F). Both metrics showed a main effect of condition (Table 1), although neither increased in the Shifting condition relative to the Baseline in either direction (Figure 3, Table 1). In contrast, the Asymmetric condition substantially increased CoP distance and ACR velocity compared to the Shifting condition (Table 1). The largest effect was in the mediolateral direction for both metrics: increasing by 123% [99%, 148%] for CoP distance and 118% [90%, 147%] for ACR velocity. In the anteroposterior direction, CoP distance increased by 32% [21%, 44%], and ACR velocity increased by 36% [21%, 52%] during the Asymmetric condition. Overall, these results confirm that asymmetric weight-bearing increases CoP variability (Figure 3B-C) and body sway (Figure 3E-F), while intermittent weight shifts did not significantly increase the variability of subsequent static postures.

### Asymmetric weight-bearing differently modulates soleus and gastrocnemius activity

**Figure 4:**
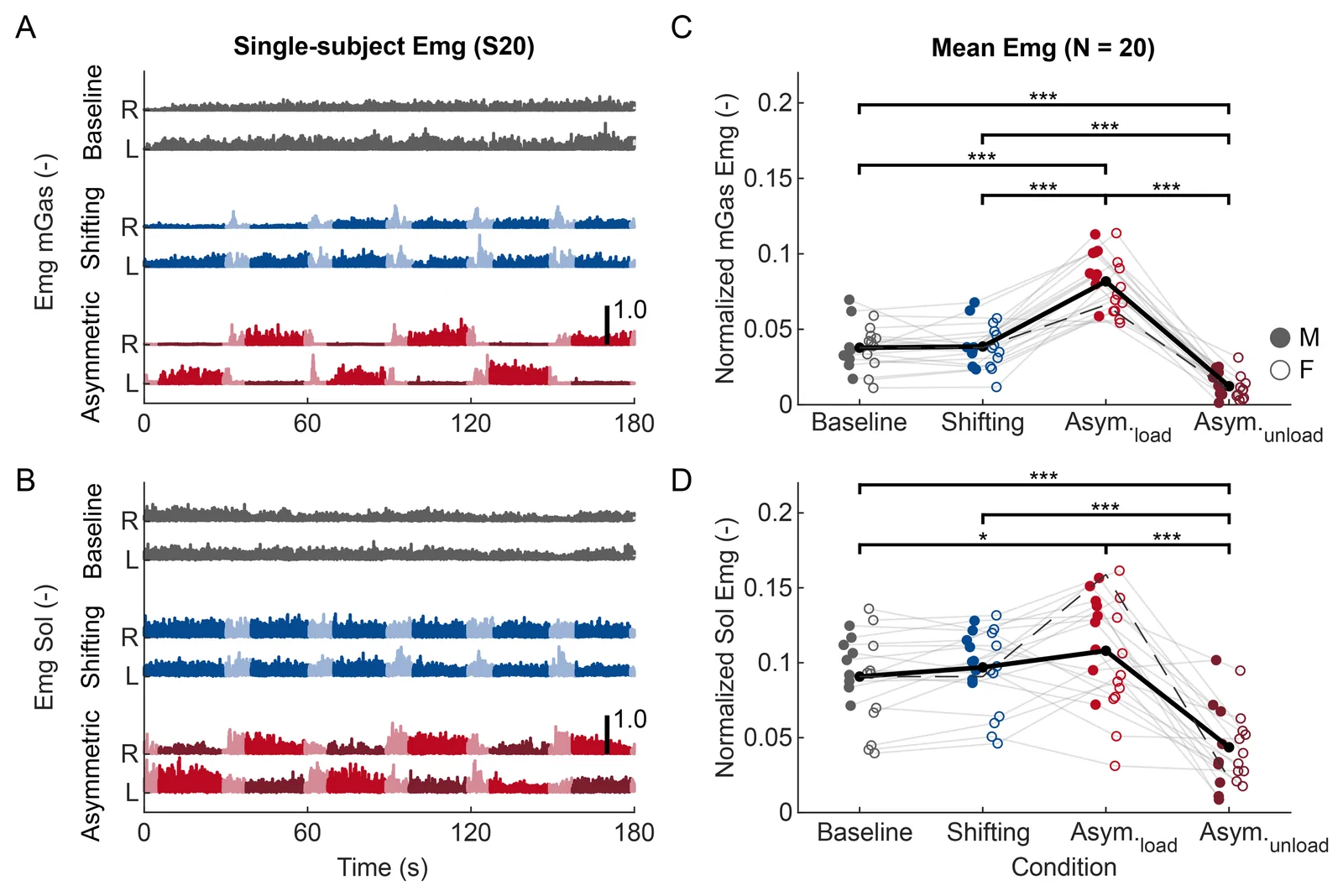
Plantarflexor EMG activity across conditions. A-B: Single-participant EMG traces for medial gastrocnemius (panel A) and soleus (panel B) across conditions. Signals during periodic weight shifts (muted color for Shifting and Asymmetric conditions) were excluded from data analysis. EMG data were normalized to the maximum observed value in that muscle throughout all trials. C-D: Group averages of muscle activity. In the Asymmetric condition, activations increased in the loaded leg (Asym._load_, bright red) and decreased in the free leg (Asym._unload_, dark red). The dashed gray line indicates the expected activation levels if activity was proportionally redistributed with a weight-bearing asymmetry of around 0.75, which would result in a 75% increase in the loaded and 75% reduction in the unloaded leg relative to Baseline. Gray connecting lines represent individual participant averages. EMG signals were normalized to the maximum observed value for each participant. Non-significant comparisons were omitted from the figure. Labels: n.s.: not significant, *: *p* ≤ 0.05, **: *p* ≤ 0.01, ***: *p* ≤ 0.001, R: Right leg, L: Left leg, M: male, F: female, mGas: medial gastrocnemius, Sol: Soleus.

Activity from both the Sol and mGas muscles varied with condition (Table 2). During the Asymmetric condition, the mGas activity approximately doubled in the load-bearing leg with respect to Baseline (difference: 116% [90%, 143%], bright-red in Figure 4A,C, Table 2) and decreased by 67% [49%, 86%] in the unloaded leg (dark-red). Sol muscle activity was also higher in the load-bearing leg as compared to the Baseline condition, though this increase was only 19% [0.2%, 38%] (Table 2, Figure 4B,D). On the other hand, the Sol activity in the unloaded leg decreased by 52% [33%, 71%]. This suggests that the Sol muscle in the load-bearing leg did not substantially increase its contribution to the plantarflexor moment in the loaded leg (see Discussion). Finally, there was also no significant difference between the Baseline and Shifting conditions for either muscle (Table 2), indicating that the increased cost of the Shifting condition (Figure 2C) could not be attributed to differences in measured muscle activity during the symmetric standing periods. We further found no difference in knee angle between Baseline and either the Shifting condition (Table 2, difference: 0.00° [-0.63, 0.62]) or the loaded leg in the Asymmetric condition (difference: 0.95° [-0.48, 2.39]), suggesting that knee angle did not confound activation patterns in these conditions. The unloaded leg was more flexed (difference: 22.3° [18.6, 25.9]) during the Asymmetric condition compared to the Baseline condition, suggesting that the unloaded limb adopted a more flexed, compliant posture.

## Discussion

In this study, we examined whether the human preference for postural weight shifting and asymmetric weight-bearing arises from a minimization of energy expenditure. Periodic weight shifts increased overall energy expenditure (Figure 2C, Baseline vs. Shifting) without significantly increasing postural variability between shifts (Figure 3). Asymmetric weight-bearing further increased the energetic cost (Figure 2C, Shifting vs. Asymmetric) and increased CoP variability and upper-body movement during the static standing phase (Figure 3). Overall, our results suggest that the common preference for weight shifting and asymmetric weight-bearing during prolonged standing is unlikely to be driven by the minimization of energetic cost and would be an ineffective strategy for doing so. Below, we discuss why humans might adopt asymmetric postures and how factors such as postural variability, muscle physiology, and biomechanics may increase energetic cost in these behaviors.

Humans tend to select postures and movements that reduce metabolic cost, yet people still spontaneously adopt asymmetric postures (1) even though this increases energetic cost (Figure 2C). On average, Asymmetric standing increased energy expenditure relative to Shifting by 0.11 W/kg [0.05, 0.16], a difference comparable to maintaining a ~1.1° more forward lean than one’s preferred posture (18) or walking roughly 0.03 m/s faster than energy-optimal (42), and is only slightly lower than the reported difference between standing and sitting (~0.16 W/kg, for review see (43)). Likewise, the estimated cost of a single weight-shift transition (2.3 J/kg [1.0, 3.6]) was comparable to walking approximately 0.75 meters (42) and to the first step of forward gait initiation (2.3 J/kg) (18). The persistence of weight shifting and asymmetric weight-bearing is notable given that behavioral energetic optimization is commonly observed in human movement, such as during symmetric standing (18) and locomotion (8–17). Even so, these cost increases were small relative to other movement costs and may therefore be secondary to other factors that drive postural selection, such as discomfort and fatigue (2–4). This supports the view that standing postural selection may reflect a trade-off in which modest increases in whole-body metabolic cost are tolerated to redistribute local loading and reduce discomfort or fatigue, rather than optimization of energy expenditure alone.

Participants in our study exhibited greater postural variability in the Asymmetric condition than in Baseline (Figure 3). It is possible that the greater variability associated with asymmetric weight bearing necessitates more frequent postural corrections, thereby increasing energy expenditure (27). This possibility is supported by other studies that increased postural demand through tandem stance (28) or a compliant surface (29), reporting a linear correlation between CoP distance and energy expenditure. Similarly, Miles-Chan et al. (30) found that variability introduced through experimentally prompted weight shifting and stepping can increase energy expenditure relative to quiet standing. Together with our observation that the Asymmetric condition exhibited greater variability (Figure 3) and energetic cost (Figure 2), these findings suggest that more frequent balance corrections in variable postures contribute to the energetic cost of asymmetric standing. This contribution may be further amplified during prolonged standing, as participants have higher postural sway when standing for longer periods (1, 5–7) and may shift their weight more frequently to alleviate accumulated discomfort or fatigue (2). Future work could assess whether weight shifts or asymmetry provide an energetic benefit in conditions where these factors play a larger role, such as during prolonged standing periods (60+ minutes) that may be encountered in occupational settings (3, 4) or when using a pre-standing condition to locally fatigue one leg (44).

At the muscle level, a key question is how asymmetric weight-bearing modulates muscle activity between the legs. We hypothesized that the plantarflexor moment, and therefore muscle activity, would be generated predominantly by the loaded leg while relaxing the unloaded leg, as this would leverage muscle physiology to reduce activation maintenance costs (21). Our EMG results (Figure 4) indicate that the gastrocnemius medialis broadly followed this pattern, with approximately double the activation of the muscle in the loaded leg and limited activity of the equivalent muscle in the free leg. However, the soleus muscle did not follow this pattern, as it only increased slightly in the loaded leg and retained around half of its Baseline activity in the unloaded leg. One possibility is that the gastrocnemius compensated for the limited increase in soleus activation by generating a larger share of the plantarflexion moment required in the loaded leg, which we observed in the loaded leg exceeding the hypothesized activation increase (Figure 4C). Such a redistribution would preserve the required ankle moment but shift force generation to a less metabolically efficient muscle, given that the gastrocnemius’s higher fast-twitch fiber ratio (45) increases its energetic cost of activation (21). Other plantarflexor muscles not measured in this study, such as the gastrocnemius lateralis and plantaris muscles, could have similarly increased their contribution. We also considered whether a change in lower-limb kinematics, specifically knee “locking” in the loaded leg, could account for the EMG pattern, but this is unlikely because the loaded leg’s knee angle did not differ significantly from that during Baseline standing. Thus, asymmetric standing appears to redistribute plantarflexor activity only partially, which may limit any potential metabolic benefit and contribute to the increased energy expenditure we observed.

Other factors not assessed in this study may also contribute to the increase in energetic cost during asymmetric standing. During symmetric standing, the pelvis is in static moment equilibrium in the frontal plane due to the symmetric vertical forces at each hip joint. When these forces are asymmetric, a hip abduction moment is required to maintain equilibrium (25, 26). While some of this moment may arise through the stretching of passive structures, this likely also imposes greater activation on muscles such as the tensor fascia latae (25), which would need to generate a continuous hip-adduction moment to maintain moment equilibrium, increasing energy expenditure. Another possibility is that the shortening-rate cost (i.e., the additional metabolic cost resulting from the contractile element changing length) increased energy expenditure in the plantarflexor muscles during Asymmetric standing. Muscle shortening costs are estimated to scale with activation to the power of 2 (as opposed to 0.6 for static activation maintenance), meaning that shortening the length of a more active muscle greatly increases its energetic cost (21). In symmetric postures, the activation maintenance cost makes up 99.4% of the energy expended by the muscle when excluding its basal energy expenditure (18). More variable asymmetric postures may increase muscle shortening costs, which do not scale advantageously with a doubling of plantarflexion moment in one leg. Furthermore, because shifts were externally prompted, the increase in energy expenditure relative to Baseline could include attention or anticipation demands, though this is unlikely to affect whole-body energy expenditure given that the brain’s energy expenditure is largely constant and independent of cognitive load (46).

Asymmetric weight-bearing can also result from bodily asymmetries rather than from a deliberate postural choice. For example, unequal leg length (47), unilateral prosthesis use (48), or hemiplegic stroke (49–51) can bias weight-bearing to one leg during standing. In contrast, leg dominance does not produce systematic asymmetries in postural control (for review, see 52). Our finding that sustained asymmetric weight-bearing carries an energetic penalty suggests that populations with pathological asymmetries may incur higher metabolic costs during standing, similar to cost increases reported for asymmetric pathological gait (53, 54). As such, it is interesting to consider whether symmetry-restoring interventions, analogous to those used in gait rehabilitation (55, 56), could also improve the energetic cost of standing balance in this population.

Sex was included as a between-subjects factor in the energy expenditure analysis to assess whether it confounded the primary energetic results. Neither the main effect of sex nor its interaction with condition reached significance (Table 1), suggesting that sex did not meaningfully confound or affect energy expenditure, though the small group sizes (9 male, 11 female) limit the confidence with which potential sex differences can be excluded. The comparison of preferred postural asymmetry suggested a potential trend toward greater asymmetry in female participants (Figure 2B), extending prior observations of a higher incidence of asymmetric postures in women (1), though this comparison was insufficiently powered and should be verified in a future study. Sex differences are generally small or absent for CoP movements recorded in lab settings (57, 58), possibly because participants are asked to adopt a symmetrical posture and therefore refrain from using asymmetric weight distributions. Future work could investigate sex differences in natural out-of-the-lab standing with larger participant groups or extend existing musculoskeletal simulations of standing balance (18, 59–62) to investigate how different muscles, particularly those around the hip joint, and individual (sex) differences affect optimal standing postures and contribute to the energetic cost of asymmetric weight-bearing.

One limitation of this analysis is that EMG signals were normalized to each participant’s maximum activity across all conditions rather than to maximum voluntary contraction (MVC). While this approach ensured consistent scaling across conditions, it did not facilitate interpretation of absolute activation levels between limbs and resulted in normalized EMG estimates higher than those normalized to MVC, which were reported to be <0.05 for mGAS and SOL during quiet standing (18). Future studies should include MVC trials to enable more direct interpretation of between-limb differences. A second potential limitation is measurement uncertainty, especially given the relatively small energetic changes. Random noise was assessed using a repeated-measures ANOVA, in which the standard error of the pairwise comparisons was less than half the observed energetic differences (see Results). This indicates that random noise had limited influence on the observed differences, regardless of whether it originated from measurement uncertainty or physiological factors. There was also no significant systematic drift across measurements (see Results), and because the condition order was randomized, any effect of drift would add to random noise rather than bias specific comparisons. The repeated-measures structure of the experiment mitigated the potential influence of fixed offsets on our main comparisons, and the measured Baseline standing energy expenditure (1.46 W/kg) fell within the range reported in previous work (1.57 ± 0.35 W/kg, converted from reported mean values in review (43)). Although our experiment was designed for within-session comparisons rather than estimating the absolute resting metabolic rate, more stringent pre-test standardization (63) in future studies could further identify the absolute energetic cost of asymmetric standing and weight shifting. Finally, weight shift trajectories differed between conditions: in the Shifting condition, participants shifted their weight laterally away and back to a symmetric posture, whereas in the Asymmetric condition, participants shifted their weight past the midline to the opposite asymmetric posture (Figure 1D-E). This means the transition movements were not fully matched between conditions, and it is possible that the asymmetric transitions incurred a slightly different energetic cost than the symmetric transitions.

In summary, we evaluated the energetic cost of two common behaviors during standing: periodic weight shifting and asymmetric weight distribution. Despite their everyday prevalence and depiction as the ideal natural posture in classical contrapposto, both behaviors increased energetic cost compared to static symmetric standing, and asymmetric weight-bearing resulted in greater postural variability. Together, these findings indicate that weight shifting and asymmetric weight-bearing are unlikely to be strategies for minimizing metabolic cost during standing and may instead serve non-energetic functions, such as alleviating discomfort or fatigue.

## Figures and Tables

**Figure 5:**
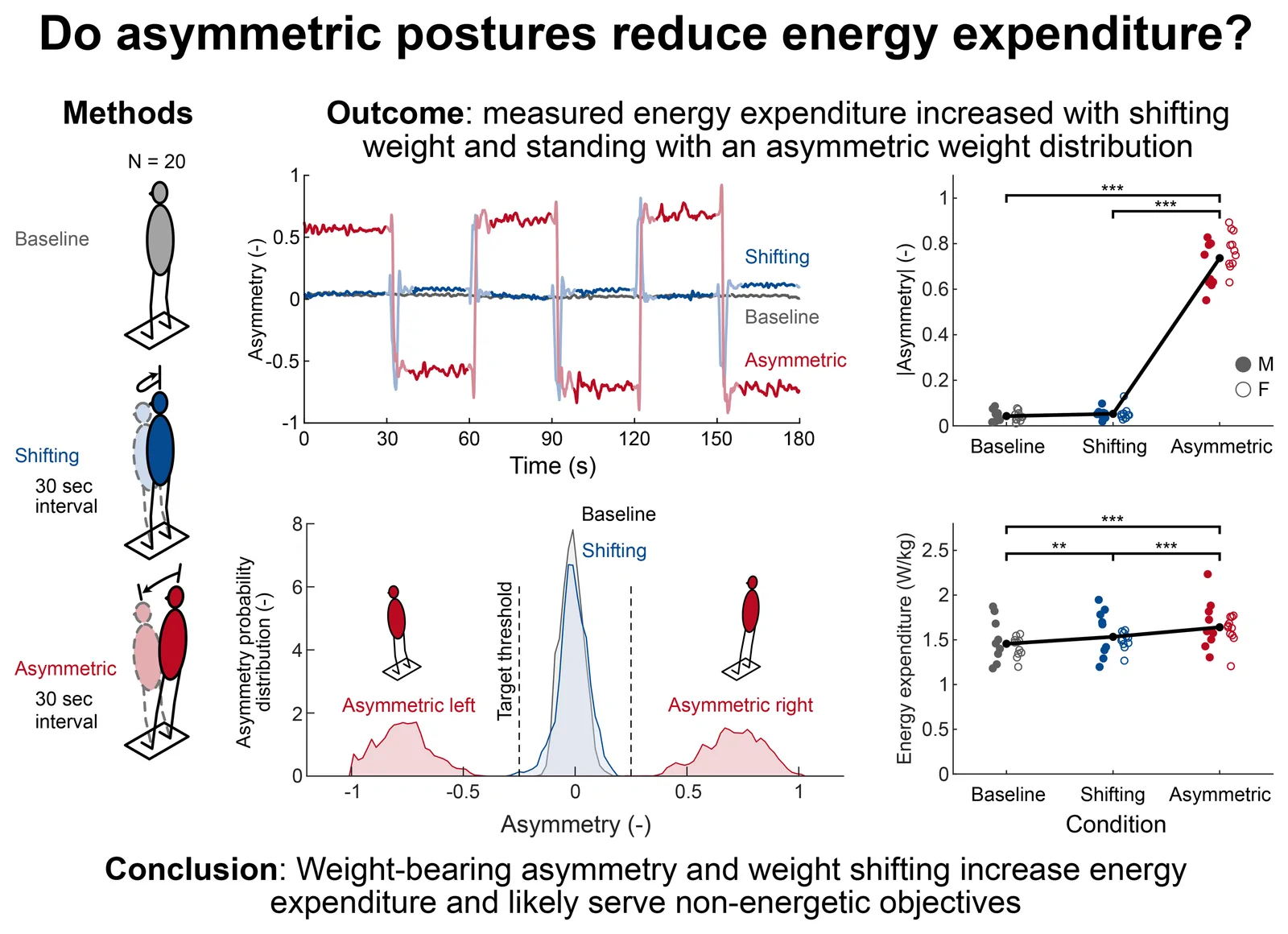


**Table 1:** Average results and ANOVA statistics for all participants (*N*=20). Energy expenditure is the mass-normalized energy expenditure measured using the metabolic analyzer. Asymmetry is the absolute value of weight-bearing asymmetry as computed in Eq. 1. CoP distance is the average distance of the CoP with respect to its mean location. Lean angle is the sagittal plane-projected angle between the approximated center of mass and the ankle joint with the vertical. ACR velocity is the mean velocity magnitude of both acromion markers in the anteroposterior (AP) and mediolateral (ML) direction. All metrics except energy expenditure exclude the periodic weight-shift phase. ^a^ Energy expenditure ANOVA included sex as a between-subjects factor; sex main effect: *F*_(1,18)_ = 0.79, *p* = 0.386; condition × sex interaction: *F*_(2,36)_ = 0.12, *p* = 0.883. ^b^ Degrees of freedom of *F* statistic: *F*_(2, 36)_; ^c^ *F*_(1.15, 21.93)_; ^d^ *F*_(1.26, 24.03)_; ^e^ *F*_(1.37, 26.08)_; ^f^ *F*_(1.43, 27.17)_; ^g^ *F*_(1.31, 24.96)_; ^h^ *F*_(1.26, 23.92)_. Labels: B: Baseline, S: Shifting, A: Asymmetric.

		Mean and SD			ANOVA		Post-hoc					
	Unit	Baseline	Shifting	Asymmetric	Condition		B - S		B - A		S - A	
					*F*	*p*	*t*	*p*_*Holm*_	*t*	*p*_*Holm*_	*t*	*p*_*Holm*_
Energy expenditure ^a^	W/kg	1.46 ± 0.18	1.53 ± 0.19	1.64 ± 0.22	24.78^b^	**<0.001**	-2.98	**0.005**	-7.01	**<0.001**	-4.03	**<0.001**
|Asymmetry|	-	0.04 ± 0.02	0.05 ± 0.02	0.74 ± 0.10	1078.7^c^	**<0.001**	-0.58	0.568	-40.51	**<0.001**	-39.93	**<0.001**
Lean angle	°	1.65 ± 0.77	1.88 ± 0.59	1.71 ± 0.71	1.14^d^	0.313	-		-		-	
CoP dist. AP	mm	3.07 ± 0.87	3.03 ± 0.81	4.00 ± 0.90	19.22^e^	**<0.001**	0.27	0.786	-5.23	**<0.001**	-5.50	**<0.001**
CoP dist. ML	mm	1.47 ± 0.55	1.73 ± 0.54	3.86 ± 0.91	121.45^f^	**<0.001**	-1.56	0.127	-14.21	**<0.001**	-12.65	**<0.001**
ACR vel. AP	mm/s	4.34 ± 1.15	4.41 ± 0.89	6.01 ± 1.96	16.999	**<0.001**	-0.21	0.837	-5.15	**<0.001**	-4.94	**<0.001**
ACR vel. ML	mm/s	2.31 ± 0.72	2.49 ± 0.66	5.44 ± 1.73	70.59^h^	**<0.001**	-0.60	0.555	-10.58	**<0.001**	-9.98	**<0.001**

**Table 2:** Average results of EMG measurements and knee angle for all participants (*N*=20). Baseline and Shifting conditions EMG are averaged over both legs. The asymmetric condition is averaged for phases where either leg is loaded (i.e., weight-bearing) or unloaded. Post hoc comparisons are reported for hypothesis-relevant contrasts between Baseline and the other conditions. Remaining pairwise comparisons were omitted as they were not motivated by a priori hypotheses. Degrees of freedom of F statistic: ^a^ *F*_(3, 57)_; ^b^ *F*_(2.18, 41.39)_; ^c^ *F*_(1.26, 22.69)_. Labels: B: Baseline, S: Shifting, A_load_: Load-bearing leg in asymmetric condition, A_unload_: Unloaded leg in asymmetric condition.

		Mean and SD				ANOVA		Post-hoc					
	Unit	Baseline	Shifting	Asymmetric		Condition		B - S		B - A_load_		B - A_unload_	
				Load	Unload	*F*	*p*	*t*	*p*_*Holm*_	*t*	*p*_*Holm*_	*t*	*p*_*Holm*_
EMG mGas	-	0.038 ± 0.015	0.039 ± 0.014	0.082 ± 0.019	0.012 ± 0.008	112.37^a^	**<0.001**	-0.21	0.837	-11.43	**<0.001**	6.63	**<0.001**
EMG Sol	-	0.091 ± 0.028	0.097 ± 0.025	0.108 ± 0.037	0.044 ± 0.026	32.05^b^	**<0.001**	-0.92	0.363	-2.70	**0.027**	6.48	**<0.001**
Knee angle	°	-2.5 ± 1.6	-2.4 ± 1.6	-3.7 ± 2.9	-25.2 ± 8.1	135.51^c^	**<0.001**	-0.07	1	0.88	1	16.71	**<0.001**

## Data Availability

All data and code supporting the findings of this study are publicly available in “Data and code for ‘Energetic cost of weight shifting and asymmetric weight-bearing during standing in humans’”, https://doi.org/10.34894/IB09YA, DataverseNL (31). Specifically, the shared data include participant averages of the metrics presented in Tables 1 and 2 (.csv), the outputs from statistical analyses performed in JASP (.pdf and .jasp), and synchronized time-series data of labeled motion-capture marker kinematics, ground reaction forces, electromyography, and metabolic analyzer data (.mat). The provided code includes MATLAB scripts for data processing and figure generation (.m).

## New and noteworthy

Natural human posture is often highly asymmetric, similar to the relaxed stance of Michelangelo’s David, and is characterized by frequent weight shifts between limbs. We experimentally tested whether such postures reduce the energetic cost of standing. Contrary to our hypothesis, participants expended more energy when standing in asymmetric postures and when shifting their weight periodically during symmetric standing, suggesting that these common postural behaviors come at an energetic expense.
